# Sandbox test setups for evaluating the performance of borehole heat exchangers: a review

**DOI:** 10.1007/s40789-026-00867-9

**Published:** 2026-02-11

**Authors:** Jian Zhao, Yunting Guo, Huawei Xu, Guangping Huang, Kourosh Gholami, Zhengmeng Hou, Wei Victor Liu

**Affiliations:** 1 Department of Civil and Environmental Engineering, University of Alberta, Edmonton, AB T6G 2E3 Canada; 2 Institute of Subsurface Energy Systems, Clausthal University of Technology, 38678 Clausthal-Zellerfeld, Germany

**Keywords:** Geothermal energy, Ground source heat pump, Borehole heat exchanger, Sandbox test

## Abstract

Geothermal energy has been increasingly gaining attention with the global transition to sustainable energy. One critical component of utilizing geothermal energy is the borehole heat exchanger (BHE), which can extract thermal energy from or inject it into the ground. Sandbox tests are often used to assess the performance of BHEs and to validate the analytical and numerical models for BHEs. However, until now, there has been no design standard for sandbox tests; a systematic review of sandbox test setups for BHE applications is notably absent from the existing literature. To address this need, a comprehensive overview was conducted for sandbox test setups used for simulating and assessing BHEs. First, the physical components of the sandbox test were introduced, including the sandbox frame, heat exchanger pipes, filling materials, seepage conditions, grouting materials, and insulation layers. In addition, the setups of the physical components of existing sandbox tests were reviewed. Furthermore, the methods to determine sandbox size and experiment duration for sandbox tests were discussed. Finally, key issues were indentified in current sandbox setups, and new insights were given to address these challenges, providing new perspectives for designing sandbox tests and thus promoting geothermal energy research.

## Introduction

To facilitate the achievement of a net-zero emission target by 2050, the use of geothermal energy has dramatically increased owing to its advantages of being remarkably stable, highly efficient, and energy-saving (Ellabban et al. 2014; Gudni 2022; Jalaluddin et al. 2011; Kubota et al. 2013; Li et al. 2015; Liu et al. 2015; Zhao and Wan 2014). According to the research by Lund and Toth (2020), the total amount of directly used geothermal energy has increased annually by 11.5% from 2015 to 2019. Within that directly used geothermal energy, the ground source heat pump (GSHP) is the most common way of harnessing this energy, accounting for approximately 71.6% of all installed geothermal capacity in 2020, and this continues to grow at an annual rate of 9.06% (Lund and Toth 2020).

The GSHP is a heat pump system that uses the ground as a heat source and sink, providing space heating, cooling, and domestic hot water for buildings (Florides and Kalogirou 2007; Shirazi 2012; Shirazi and Bernier 2014; Sliwa et al. 2016; Soni et al. 2015, 2016; Yan and Xu 2018). This system commonly operates as a closed loop, utilizing the borehole heat exchanger (BHE) to facilitate heat exchange between the ground and the heat pump. The typical BHE system contains a group of buried pipes installed in the vertical boreholes at depths between 50 and 150 m, in which the working fluid is circulated to transfer heat (Ahmadfard and Bernier 2019; Benli 2013; Cui et al. 2011; Hengel et al. 2020; Huang et al. 2020; Huttrer 2020; Lucia et al. 2017; Sarbu and Sebarchievici 2014; Toth and Bobok 2017). During heating operations, the GSHP system extracts heat from the ground and injects it into the building, while during cooling operations, heat is extracted from the building and injected into the ground through BHEs (Florides and Kalogirou 2007). The performance of BHEs is crucial to the efficiency of a GSHP system, as it determines the amount of thermal energy that can be extracted from the ground (Guo et al. 2024b). Therefore, it is essential to properly evaluate the thermal performance of BHEs and optimize their design to ensure they meet the energy demands of the buildings they serve.

To understand the thermal performance of BHEs and optimize their design, researchers have developed various analytical and numerical models (Al-Khoury et al. 2005; Bauer et al. 2011a, 2011b; Bouhacina et al. 2015; Choi et al. 2013; Claesson and Eskilson 1988; Diersch et al. 2011a, 2011b; Eskilson and Claesson 1988; Holmberg et al. 2016; Lazzari et al. 2010, 2012; Minaei and Maerefat 2017a, 2017b; Rees and He 2013). Analytical models normally rely on several simplified assumptions, such as regular geometries, homogeneous and temperature-independent thermal properties, and perfect thermal contacts between the pipe, grout, and ground (Guo et al. 2023, 2024a; Li and Lai 2015). While they make analytical models quicker and easier to evaluate the performance of BHEs, these assumptions have ignored real-world complexities like heterogeneous ground conditions, phase-change grouting materials, and imperfect interfaces. On the other hand, numerical models can better handle irregular geometries, complex ground conditions, and intricate behaviors of phase-change materials, providing a more accurate solution than analytical models (Badenes et al. 2020; Javadi et al. 2022; Zhao et al. 2024a). However, numerical models require significant computational resources and time, especially for large-scale or high-resolution simulations (Guo et al. 2024a). Crucially, both analytical and numerical models require validation to ensure their predictions align with real-world observations. This typically involves comparing model outputs with experimental data or field monitoring results.

To accurately assess the performance of BHEs under real-world conditions, and to validate analytical and numerical models, experimental data or field monitoring results are essential. A few field monitoring results have been reported in conjunction with in-situ Thermal Response Test (TRT) to estimate the effective thermal properties of the ground (Gehlin 2002; Li and Lai 2015; Raymond et al. 2009). However, these reported field monitoring results typically cover only a few days and lack the comprehensive information to fully characterize the complex subsurface condition. In addition, conducting full-scale field tests is resource-intensive, requiring significant resources (e.g., proper test sites and specialized equipment) and logistics, making them costly and time-consuming, especially for long-term operations. In contrast, the sandbox test offers several advantages. First, using a smaller-scale rig to replicate the BHE system, the sandbox test eliminates the need for large test fields and drilling operations, significantly reducing both time and cost, even for long-term experiments (Cimmino and Bernier 2015). Additionally, the sandbox test allows for the replication of complex material behaviors and interactions between system components in a controlled environment (Javadi et al. 2022). This controlled setting enables more precise isolation and examination of key parameters influencing BHE performance, making it easier to adjust and replicate test conditions across different scenarios (Baker et al. 1982; Fan et al. 2013; Guo et al. 2020; Guo et al. 2021; Pimentel et al. 2012; Zhao et al. 2014; Zhao et al. 2022). By simulating the BHEs, the sandbox test closely resembles analytical models that allows for a thorough and complete model validation (Ghasemi-Fare and Basu 2016; Tinti et al. 2015; Zhang et al. 2015). Moreover, the experimental data gathered from the sandbox test can be used to verify numerical models, making these tests essential for improving simulation accuracy (Cai et al. 2019; Cimmino and Bernier 2015; Kong et al. 2017; Mousa et al. 2020; Pu et al. 2015). In brief, offering a balance between experimental control, cost-efficiency, and detailed data, the sandbox test presents a highly effective method for investigating BHE performance, ultimately contributing to better geothermal energy utilization and supporting sustainable development.

Yet, despite the existing advantages mentioned above, there are few sandbox tests reported in the literature. Moreover, the wide variation in the setups of physical components among reported sandbox tests fundamentally affects the comparability and reproducibility of the experimental data used to evaluate BHE performance. Until now, there are no established guidelines for conducting sandbox tests, particularly with regard to the setup of the physical components. This lack of guidelines impedes the development and implementation of sandbox tests, leads to inadequate evaluation of the critical impacting factors in the assessment of the BHEs’ performance, and ultimately results in the inefficient application of geothermal energy. Therefore, a comprehensive review of sandbox tests is highly desirable to consolidate the physical component setups required to accurately simulate the performance of BHEs. This review paper provides researchers with guidelines on carrying out sandbox tests and deepens understanding of key factors affecting the performance of BHE systems. To this end, the objective of this work is to present an introduction and comprehensive guide to the physical component setup to simulate the performance of BHEs by sandbox tests.

The paper is structured as follows: Section 2 presents the apparatus and methodology of sandbox tests. Section 3 summarizes the setup of the physical components of sandbox tests, including the sandbox frame, filling materials, grouting materials, heat exchanger pipes, circulating fluid, thermocouple, groundwater seepage, and insulation layer. Section 4 provides guidance to design the dimensions and experiment duration of the sandbox tests. Section 5 presents a summary of the existing problems and perspectives of reported sandbox tests. Finally, the concluding remarks are presented in Section 6.

## Overview of sandbox tests

### Sandbox test rig

**Fig. 1:**
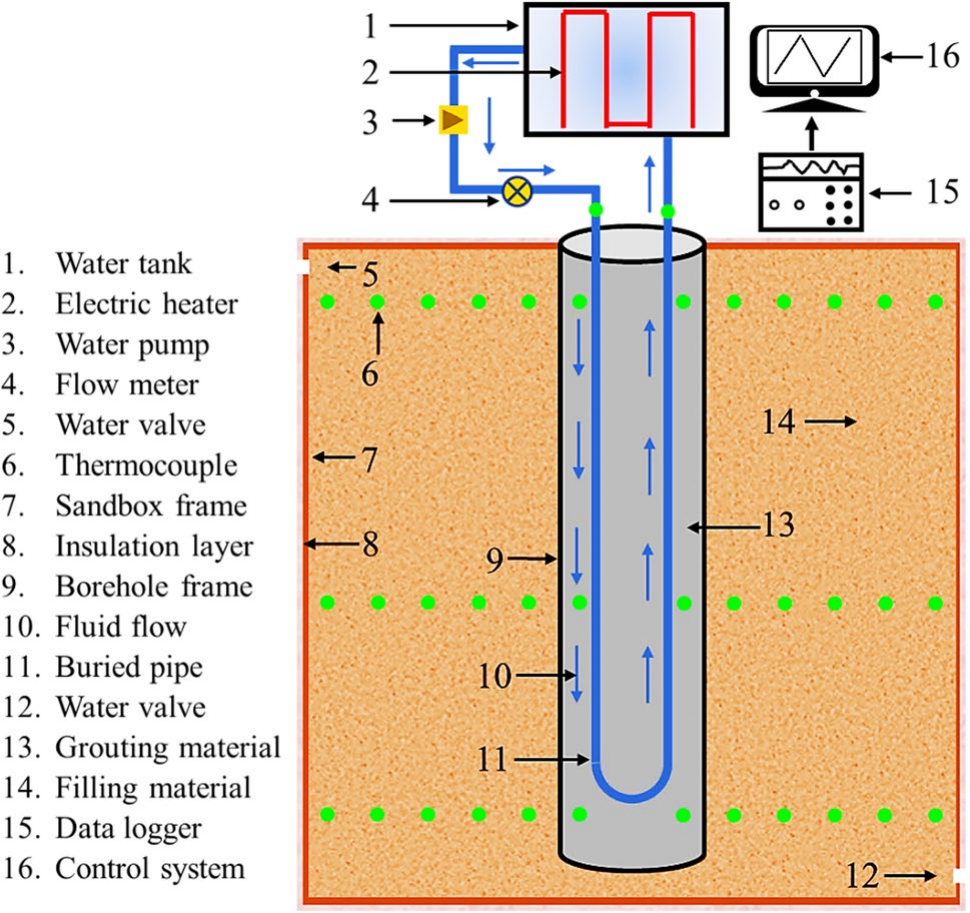
Schematic configuration of sandbox test rig

To conduct the sandbox test, several apparatus and physical components are commonly installed and assembled, including the (1) water tank, (2) electric heater, (3) water pump, (4) flow meter, (5) water valve, (6) thermocouple, (7) sandbox frame, (8) insulation layer, (9) borehole frame, (10) circulating fluid, (11) heat exchanger pipe, (12) water valve, (13) grouting material, (14) filling material, (15) data logger, and (16) control system, as illustrated in Fig. 1. The sandbox frame accommodates the testing apparatus and physical components that represent the portion of the BHE system buried in the ground, while the borehole frame is installed for casting the grouting material. The filling material within the sandbox frame is used to replicate subsurface conditions. The water pump and flow meter are employed to regulate the water flow rate inside the pipe, and the electric heater are used to control the water temperature inside the water tank. The data logger coupled with the control system is applied to collect the experimental results. When seepage conditions are simulated in the sandbox test, an additional water reservoir and valves can be used to control the seepage velocity, thereby reproducing various groundwater flow scenarios.

### Sandbox test methodology

To conduct the sandbox test, the sandbox frame is first assembled and positioned. The filling material (e.g., sand and clay) is then placed within the sandbox frame to reproduce the subsurface conditions, and a set of thermocouples are strategically placed at specific locations within the filling material. Meanwhile, heat exchanger pipes, equipped with thermocouples, are placed in a borehole installed in the filling material. The space between the pipes and the borehole wall can be grouted. All thermocouples are connected to the data logger, which is linked to the control system. Finally, the heat exchanger pipe are connected to the water pump, flow meter and water tank.

During the test, the water pump circulates fluid through the heat exchanger pipe at a constant flow rate. The circulating fluid entering the pipe is heated by the electric heater. In sandbox tests, it is common practice to either maintain a constant fluid inlet temperature or operate the electric heater at a fixed power output. The temperatures of the fluid, borehole wall, and filling material are continuously measured by thermocouples and are recorded by the data logger. The actual injected heat is determined by comparing measured fluid temperatures at the inlet and outlet of the heat exchanger pipe. The thermal performance of the BHE system is further evaluated by analyzing the temperature distribution in the filling material.

## Setups of physical components

This paper focuses on the setups of the physical components within the sandbox frame, including the sandbox frame, filling material, grouting material, heat exchanger pipe, circulating fluid, thermocouple, groundwater, and thermal insulation layer. Table 1 summarizes the reported sandbox tests in terms of simulating the performance of BHEs.

**Table 1:** Summary of the reported sandbox tests on BHEs

References	Pipe shape, material, direction	Sandbox shape, size (m), material	Inlet flow rate (ml/s), temperature (˚C)			Filling material	Grouting material	Groundwater seepage	Initial sandbox temperature (˚C)	Sandbox insulation
Shirazi et al. (2012, 2014)	U-tube copper vertically	Cylindrical H × D = 1.35 × 1.4 HDPE	FR: 11.4 ml/s TEM: 67.5 °C			Ottawa sand	NA	NA	23.5 °C	☑
Cimmino et al. (2015)	U-tube copper vertically	Cylindrical H × D = 1.35 × 1.4 HDPE	FR: 0.502 ml/s TEM: 70 °C			Ottawa Sand	NA	NA	23 °C	☑
Yu et al. (2004, 2008)	U-tube HDPE vertically	Cubic L × W × H = 2.5 × 1.25 × 1.8 steel	FR: (ml/s) Pre-storage: 87 Charging: 86 Discharging: 108 TEM: 10 °C			Sand (moisture 8.61%)	NA	NA	10 °C	☑
Zhang et al. (2020)	U-tube PPR vertically	Cubic L × W × H = 0.6 × 0.6 × 1.2 steel	FR: 265 ml/s TEM: (˚C) Heat injection: 40, 45, 50 Heat extraction: 3, 4, 5			Sand	NA	NA	15 °C (heat injection) 18 °C (heat extraction)	☑
Zhang et al. (2022)	U-tube PPR vertically	Cubic L × W × H = 0.6 × 0.6 × 1.2 steel	FR: 222.6 ml/s TEM: 35 °C			Sand, fine sand, glass beads (equal thickness: 0.4 m)	NA	NA	17 °C	☑
Reuss et al. (1997)	Coaxial – vertically	Cylinder H × D = 1 × 1	FR: – TEM: Charging: 25 °C Discharging: 50 °C			TE1: silica sand (moisture 7.5%) TE2: opalinus clay (moisture 7%) TE3: Kaolin clay (moisture 30%)	NA	NA	–	–
Li et al. (2017, 2018)	U-tube copper horizontally	Cubic L × W × H = 6.25 × 1.5 × 1 concrete	FR:12.76 ml/s TEM: 29.3 °C			Sand, Clay	NA	NA	13.8 °C	☑
Li et al. (2019)	U-tube copper horizontally	Cubic L × W × H = 6.25 × 1.5 × 1 concrete	FR: (ml/s) TT1:12.76 TT2:12.76 TT3:14.52 TT4:12.76	TEM:(˚C) TT1: – TT2: 12.5 TT3: 15 TT4: 15		Sand, Clay	NA	NA	TT1: 13.85 °C TT2: 12.23 °C TT3: 10.17 °C TT4: 17.17 °C	☑
Wang et al. (2018, 2013)	U-tube HDPE vertically	Cylindrical H × D = 0.9 × 0.8 steel	FR: – TEM: below 0 °C			Soil (well-saturated)	NA	NA	4 °C	☑
Eslami Nejad et al. (2012, 2011)	Single-pipe copper vertically	Cylindrical H × D = 0.5 × 0.43 PVC	FR: – TEM: Case3: -20 °C			Ottawa Sand TE2: dry sand TE3: saturated sand with freezing	NA	NA	Case2: 18.3 °C Case3: 23.5 °C	☑
Kramer et al. (2015)	U-tube PVC vertically	Cubic L × W × H = 1.83 × 1.83 × 0.91 steel	FR: 13.34 ml/s TEM: 39 °C			Ottawa Sand	NA	NA	19 °C	☑
Jin et al. (2020)	Single-pipe copper vertically	Cubic L × W × H = 1.3 × 1.3 × 1.5 steel	FR: – TEM: 26.85 °C			Sand	NA	NA	14.85 °C	☑
Beier et al. (2011)	U-tube HDPE horizontally	Cubic L × W × H = 18 × 1.8 × 1.8 wood	FR: 197 ml/s TEM: 23 °C			Sand (saturated)	NA	NA	22 °C	–
Li. et al. (2020)	U-tube copper horizontally	Cubic L × W × H = 6.25 × 1.5 × 1 concrete	FR:(ml/s) TT1:12.76 TT2:12.76 TT3:12.56 TT4:12.76	TEM:(˚C) TT1: – TT2: – TT3: 32 TT4: 32		Sand, Clay	NA	TT2:1.11(m/d) (TEM:23.4 °C) TT4:0.94(m/d) (TEM:25.1 °C)	TT1: 15.4 °C TT2: 24.0 °C TT3: 20.4 °C TT4: 24.2 °C	☑
Ma et al. (2021)	Single-pipe PPR vertically	Cubic L × W × H = 1.2 × 0.8 × 1.2 Plexiglas	FR: – TEM: 16 °C			Sand	NA	Darcy velocity 2.4–3.2 × 10^–7^ to 2–3 × 10^–6^ m/s	18 °C	☒
Erol et al. (2014, 2013)	Single-pipe HDPE vertically	Cubic L × W × H = 1 × 1 × 1 wood	FR: 366 ml/s TEM: Heat pump1: 12 °C Heat pump2: 15 °C			Sand (case1: dry, case2: saturated)	TE1: silica sand-based TE2: bentonite-based TE3: graphite-based	NA	20 °C	☑
Yang et al. (2019)	U-tube copper vertically	Cubic L × W × H = 1.2 × 1.2 × 1.2 wood	FR: – TEM: 30–37 °C (summer mode), 5–10 °C (winter mode)			Sand	TE1: Decyl acid and lauric acid (summer mode) TE2: oleic acid (winter mode)	NA	35 °C (summer mode) 5 °C (winter mode)	☑
Javadi et al. (2022)	Single-pipe PE vertically	Cubic L × W × H = 1 × 1 × 1 wood	FR: 500 ml/s TEM: Single-column test: one cycle: heat injection 20 °C → 40 °C → 50 °C, Cooling down 50 °C → 20 °C Four-column test: one cycle: Heat injection 20 °C → 50 °C, cooling down 50 °C → 20 °C			Sand (well saturated)	TE1: enhanced grout TE2: mixture of enhanced grout and MPCM TE3: mixture of enhanced grout and SSPCM	NA	20 °C	☑
Gu et al. (1998)	U-tube cooper vertically	Cylindrical H × D = 1.16 × 0.8 –	FR: 6.67 ml/s TEM: TT-A Phase1: 22.2–55 °C Phase2: 55–40.7 °C Phase3: 40.7–53.4 °C Phase4: 53.4–39.6 °C Phase5: 39.6–56.6 °C TT-B Phase1: 22.2–63.7 °C Phase2: 63.7–38.7 °C Phase3: 38.7–56.2 °C Phase4: 56.2–40.1 °C Phase5: 40.1–60.9 °C			Soil (moisture 14%)	TE1:12.5% bentonite, 25% sand, 62.5% water TE2: 10% bentonite, 25% copper powder, 65% water	NA	22.5 °C	☑
Wang et al. (2023)	U-tube HDPE horizontal	Cubic	0.6 m/s 39.85 °C			Soil (saturation: 0.1–0.9)	NA	1 × 10^–7^–1 × 10^–5^ m/s	9.85–17.85 °C	☑

### Sandbox frame

**Fig. 2:**
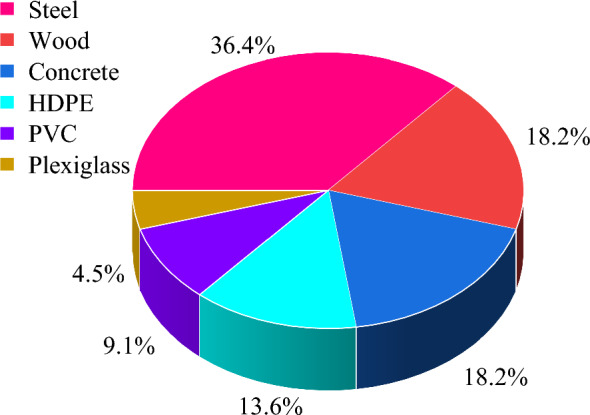
Materials used for constructing sandbox frames

The sandbox frame is a pre-manufactured structure designed to facilitate the installation and placement of the other physical components (e.g., the filling material) in sandbox tests. Several factors are usually considered in the design of the sandbox frame, including its shape, materials, size, and interior structure. As listed in Table 1, cubic and cylindrical frames are the two main shapes of the sandbox. Different types of materials can be used to construct the sandbox frame, including steel, wood, concrete, high-density polyethylene (HDPE), polyvinyl chloride (PVC), and plexiglass, as presented in Fig. 2.

The size of the sandbox frames depends on the designed test duration and the space capacity of the laboratory. According to the reported sandbox tests, typical dimensions for cubic frames are less than two meters in length, width, and depth, and they are also less than two meters in height and diameter for cylindrical frames, as summarized in Table 1. It is worth noting that some researchers, by orienting heat exchanger pipes horizontally, employed longer boreholes and frames. For instance, Yu et al. (2008) extended the length of the sandbox to 2.5 m to assess the performance of a U-shaped pipe laid in three layers. Similarly, Li et al. (2018) used a sandbox with a depth of 6.25 m and a width of 1.5 m to evaluate the performance of a U-shaped pipe installed in a multilayered backfill. Besides, Beier et al. (2011) conducted the sandbox tests with a medium-scale sandbox frame which measured 18 m in depth and 1.8 m in both width and length. This enables researchers to evaluate the performance of a large borehole with a depth of 18 m and diameter of 12.6 cm, which resemble the actual dimensions of a BHE. Although orienting heat exchanger pipes horizontally allows for longer boreholes without being limited by the height of the space, it may limit the radial distance from the heat exchanger pipes to the frame boundaries. If this distance is insufficient, the frame boundaries can significantly influence the temperature distribution within the sandbox, especially in the later stages of the experiment. This issue will be discussed in detail in Sect. 4.

**Fig. 3:**
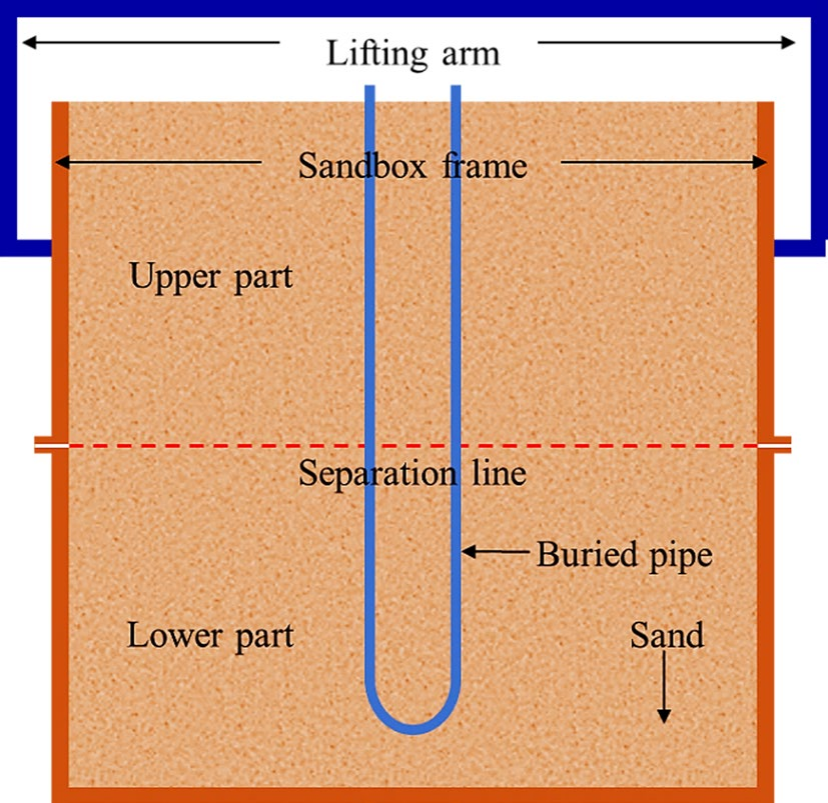
Schematic configuration of separated sandbox frame (modified from Kramer et al. (2015))

In the reported sandbox tests, the typical structure of the sandbox frame consists of a single, undivided frame. Notably, Kramer et al. (2015) introduced a modification to the frame by separating it into two parts: an upper part and a lower part, as illustrated in Fig. 3. The two parts can be securely connected using bolted joints around the frame. This two-part frame structure allows for easier access to the lower part, without the upper part in place, during the filling of the sand and the installation of instruments.

**Fig. 4:**
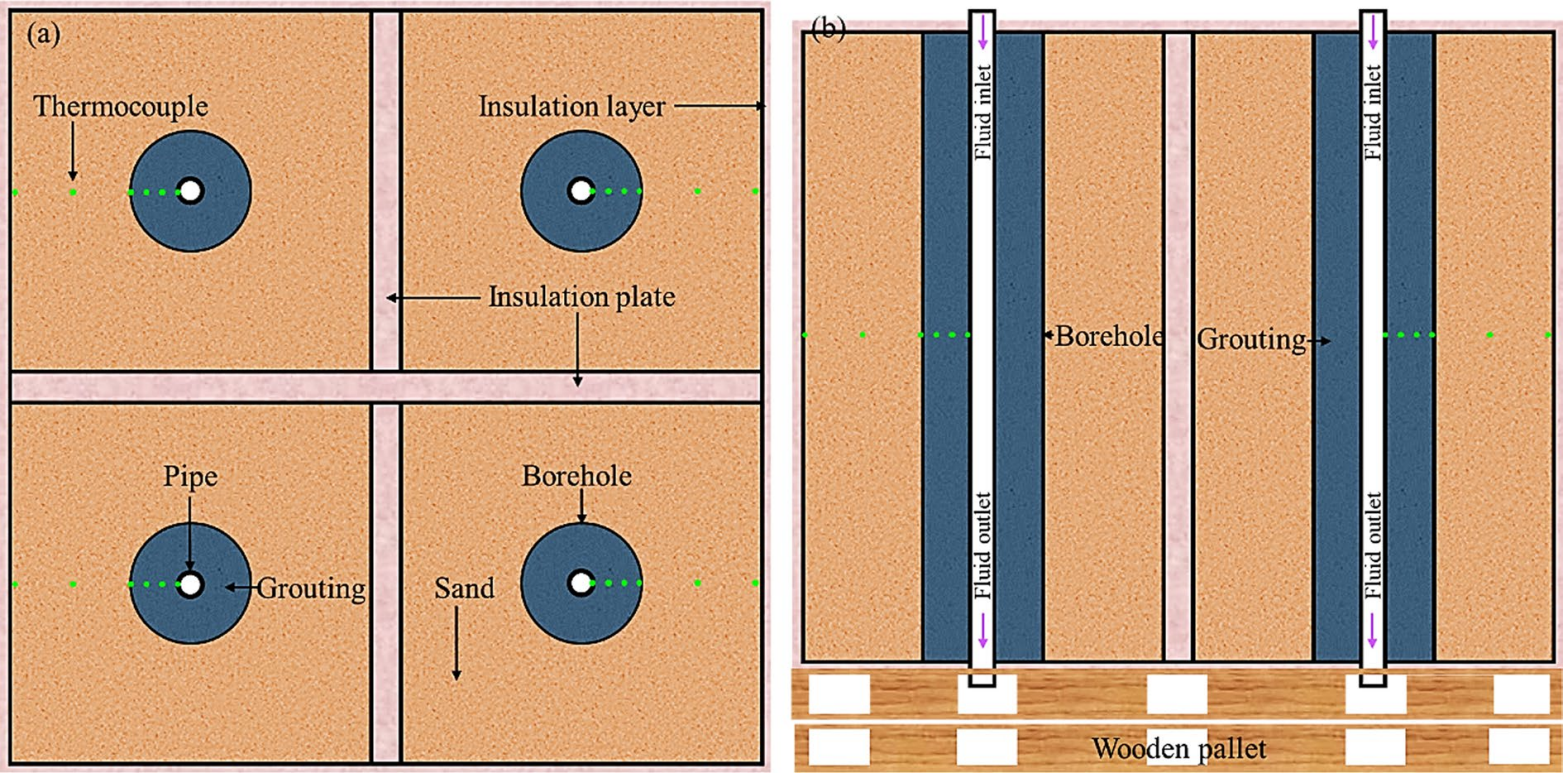
Sandbox test frame for grouting material testing (modified from Javadi et al. (2022))

Although the sandbox with a single structure is sufficient to evaluate the performance of BHEs in most cases, researchers have developed complex structures to explore specific scenarios. For example, Javadi et al. (2022) proposed a complex frame by dividing the entire sandbox frame into four identical parts, as depicted in Fig. 4, to evaluate the performance of grouts in BHEs. Utilizing this sandbox frame enables four different grouts to be evaluated simultaneously during a single test, thereby enhancing the efficiency of the testing process.

### Filling material

The filling material is one of the main components of the sandbox which reproduce the ground where BHEs are installed. Commonly used filling materials in sandboxes include sand and clay, as summarized in Table 1. These materials are natural, common materials that can be easily found to simulate the actual ground surrounding the BHEs. Other artificial materials, such as glass beads, were also used as filling materials in a previous study (Zhang et al. 2022).

In sandbox experiments, the filling material is commonly planned to be homogeneous, meaning that the density, moisture content, thermal conductivity, volumetric heat capacity, and thermal diffusivity of the filling material remain consistent throughout the sandbox (Cimmino and Bernier 2015; Zhang et al. 2022). This approach enhances the repeatability of the experiments and facilitates cross-validation with analytical models. Meanwhile, weakening the influence of material heterogeneity allows for a focused investigation of other key factors affecting BHE performance. To achieve homogeneous properties of the filling material, researchers have implemented several strategies. First, the use of standard sand or clay is prevalent due to their well-documented characteristics, including thermal properties, particle size distribution, and void ratio. For example, Ottawa sand, a graded pure quartz sand with well-tested thermal properties (Tarnawski et al. 2009), is often recommended based on previous studies (Cimmino and Bernier 2015; Kramer et al. 2015). Second, the placement of the filling material is critical for achieving homogeneous properties. Tamping the sand or clay to make it compact is a common method used to homogenize it. Additionally, Kramer et al. (2015) developed a sand placement method called “sand-raining”, which uses a pluviation system to “rain” dry sand into the sandbox frame. The pluviation system consists of a perforated box with a controlled opening filled with sand and up to four layers of sieves with different sizes at the bottom of the box to spread the falling sand uniformly. Through this system, the density of sand in the sandbox can be controlled by the height of the perforated box and the number and size of the sieves.

**Fig. 5:**
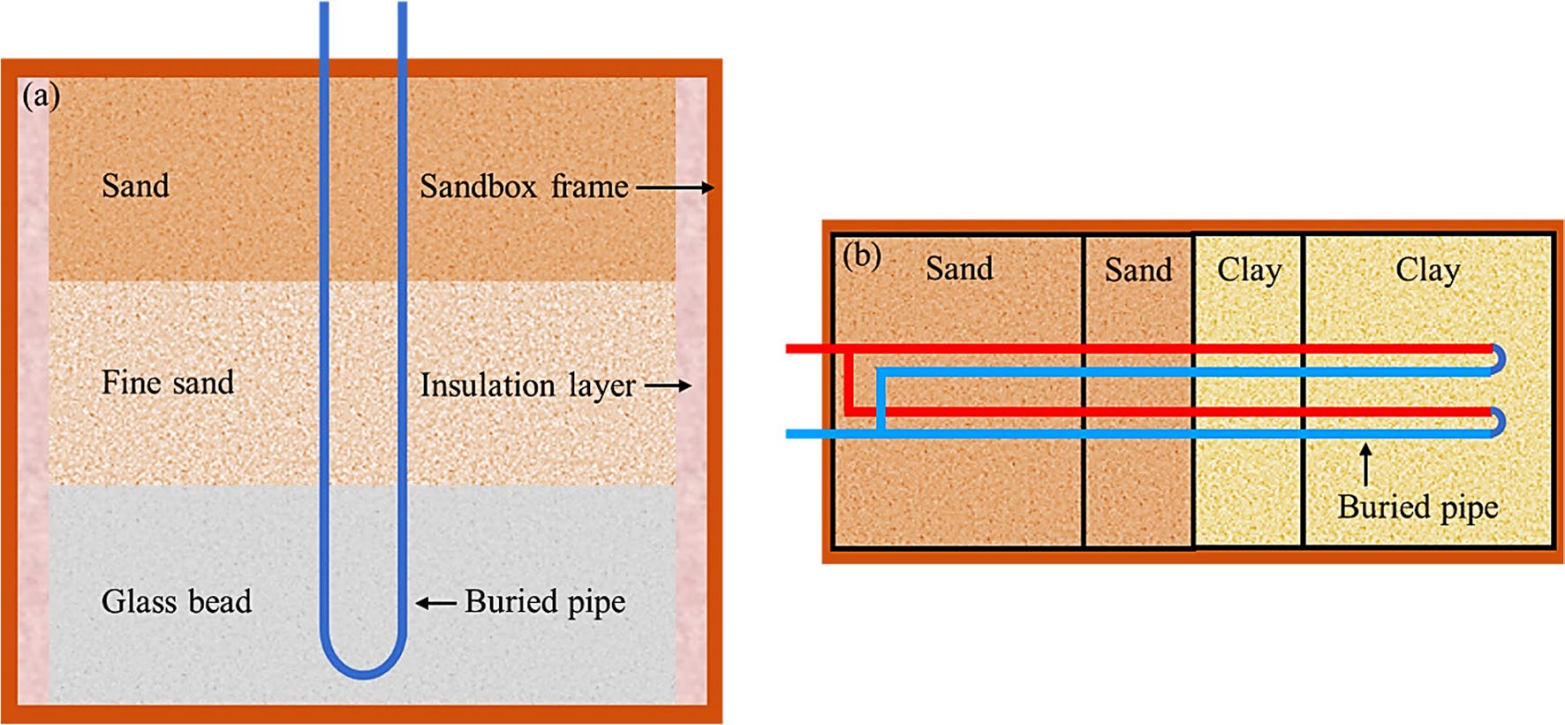
Schematic configuration of layered subsurface in the sandbox (modified from Zhang et al. (2022) and Li et al. (2020))

Most of the reported sandbox tests employed one single type of filling material to study its thermal behavior, as presented in Table 1. However, the actual BHEs are typically installed in layered ground. Therefore, two or three different filling materials can be used to reproduce the layered ground to better understand the performance of BHEs in such environments. For example, Zhang et al. (2022) employed three different filling materials—well-measured laboratory sand, fine sand, and glass beads—to simulate the layered ground, as depicted in Fig. 5a. Li et al. (2018, 2020) used the laboratory’s well-sieved local sand and clay in sandbox tests, in which the sandbox frame was separated into four sections to develop the layered subsurface, as presented in Fig. 5b.

### Grouting material

The grouting material is utilized to fill the gaps between the heat exchanger pipe and the ground, and it performs as the medium for heat exchange between the heat exchanger pipe and the ground (Zhao et al. 2024b). In addition, the grouting material protects the structural integrity of the BHE by forming a permanent seal in the borehole (Bergen et al. 2022; Bett 2017; Blazquez et al. 2017; Desmedt et al. 2012; Indacoechea-Vega et al. 2018; Kurevija et al. 2017; Liu et al. 2021a; Mahmoud et al. 2021; Naldi et al. 2021; Pascual-Muñoz et al. 2018; Pyatina and Sugama 2020; Sliwa and Rosen 2017). From the reported sandbox tests on the grouting material, the selection criteria for the grouting material are: (1) a high thermal conductivity; and (2) a good flowability so that they can be placed easily into the borehole. Currently, there are two main types of grouting materials: bentonite-based grouts (Delaleux et al. 2012; Kim et al. 2015; Liu et al. 2019) and cement-based grouts (Allan and Philippacopoulos 1999; Dong et al. 2022; Zhao et al. 2024a, 2024b, 2025). Recently, the use of phase change material (PCM) in the grouting material was reported because of its ability to store and release thermal energy during the process of melting and solidification (Bernier 2013; Faraj et al. 2020; Javadi et al. 2022; Joulin et al. 2011; Liu et al. 2021b; Lyne et al. 2019; Mahmoud et al. 2021; Mousa et al. 2021; Pássaro et al. 2022; Qi et al. 2016; Shukla et al. 2009; Zalba et al. 2003).

When conducting sandbox tests, grout-pipe samples can be cast in a temporary mould (e.g., a cardboard mould) in advance outside the sandbox. In this process, the thermocouples can be embedded in the grouts to monitor the temperatures. After curing the grouts for 28 days, grouts-pipe samples can be positioned and placed in the sandbox frame with the filling material. Once each test is completed, the grouting material and the pipe are removed from the sandbox frame. Then, the sandbox test components and apparatus are reinstalled to test another grouting material.

However, this installation-removal-installation procedure with the components and apparatus can easily cause different testing conditions of the physical components between tests. This procedure also makes the testing of grouting materials time-consuming. To mitigate the differences in testing conditions caused by the installation-removal-installation procedure, Javadi et al. (2022) proposed a sandbox frame to set up the grouting material, as depicted in Fig. 4. Four different types of grouting materials can be installed and examined simultaneously during one test by employing this sandbox frame. In addition, the performance of the same grouting material under various working conditions can be achieved in one test by changing the properties of the filling materials in each section of the sandbox frame.

### Heat exchanger pipe

The heat exchanger pipe is used to circulate the heat carrier fluid to complete the heat exchange process with the ground. In the previous sandbox tests, three shapes of heat exchanger pipes were used, including the U-shaped pipe, single straight pipe, and coaxial-shaped pipe, as summarized in Table 1. Most previous studies adopted the U-shaped pipe in sandbox tests since it is the most commonly used type of pipe in actual BHEs (Cimmino and Bernier 2015; Li et al. 2018, 2020; Zhang et al. 2020). To maintain the constant spacing between the two branches of a U-shaped pipe along the borehole, polyphenylene oxide (PPO) spacers can be installed along the pipe (Cimmino and Bernier 2015). The single straight pipe has also been used in reported sandbox tests as a simplification of the U-shaped pipe when the effect of the pipe shape can be neglected (Erol and Francois 2014; Erol and François 2013; Javadi et al. 2022; Ma et al. 2021; Yu 2004; Yu et al. 2008). For example, Javadi et al. (2022) installed a single pipe in the PCM-based grouts to investigate the thermal performance of grouts when they are used in BHEs or borehole thermal storage systems, as depicted in Fig. 4. Notably, when using a single straight pipe for heat exchanging, a hole should be reserved at the bottom of the sandbox frame for the outflow of water.

In actual BHEs, the diameter of the heat exchanger pipe typically ranges from 2 to 4 cm (Gudni 2022; Li et al. 2014b; Rybach 2001, 2022; Zanchini and Jahanbin 2018). Although most reported sandbox tests have scaled down the pipe length, the diameter of the pipe was rarely scaled accordingly, as summarized in Table 2. Consequently, the test results may not accurately reflect the three-dimensional heat distribution in real-world applications. Only a few studies have considered reducing the pipe diameter. For instance, Cimmino and Bernier (2015) scaled down the pipe diameter to 3.715 mm, preserving the aspect ratio.

**Table 2:** Summary of the heat exchanger pipe diameter

References	Pipe diameter
Shirazi et al. (2012, 2014)	9.5 mm (outer diameter), 7.9 mm (inner diameter)
Erol et al. (2014)	32 mm (outer diameter), 26.2 mm (inner diameter)
Cimmino et al. (2015)	3.175 mm (outer diameter), 1.651 mm (inner diameter)
Zhang et al. (2020)	20 mm (outer diameter), 15 mm (inner diameter)
Yu et al. (2004, 2008)	15 mm (nominal diameter, Study 1), 20 mm (nominal diameter, Study 2)
Zhang et al. (2022)	20 mm (outer diameter), 15 mm (inner diameter)
Li et al. (2017; 2018)	11 mm (outer diameter), 10 mm (inner diameter)
Li et al. (2019; 2020)	11 mm (outer diameter), 10 mm (inner diameter)
Wang et al. (2018; 2013)	32 mm (outer diameter), 25 mm (inner diameter)
Eslami Nejad et al. (2012, 2011)	22 mm (outer diameter)
Kramer et al. (2015)	15.8 mm (outer diameter), 12.4 mm (inner diameter)
Beier et al. (2011)	33.4 mm (outer diameter), 22.73 mm (inner diameter)
Yang et al. (2019)	6.5 mm (outer diameter), 5 mm (inner diameter)
Javad et al. (2022)	20 mm (outer diameter), 16 mm (inner diameter)
Gu et al. (1998)	6.4 mm (outer diameter)

**Fig. 6:**
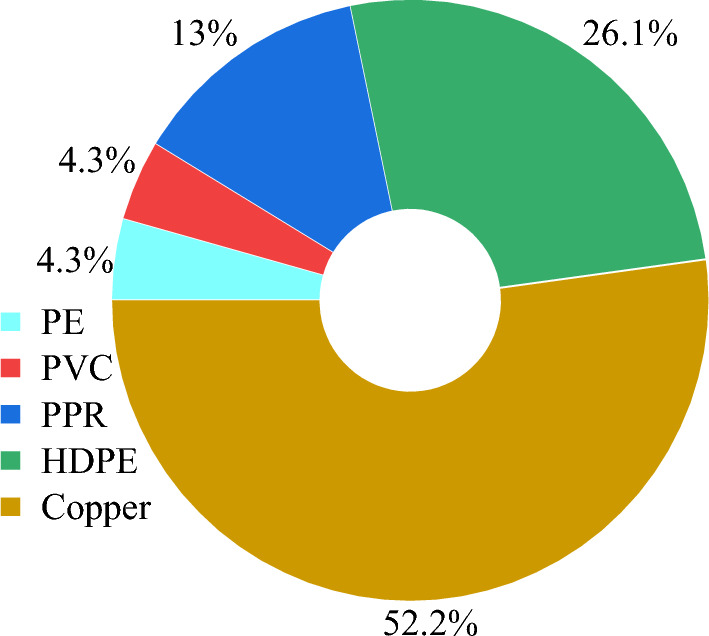
Materials used for fabricating heat exchanger pipes

In the reported sandbox tests, the heat exchanger pipe was made of either metal or non-metal materials. Typically, copper is used as the metal material for pipes. The non-metal material includes high-density polyethylene (HDPE), polypropylene random copolymer plastic (PPR), polyethylene (PE), and polyvinyl chloride (PVC), as illustrated in Fig. 6. In actual BHEs, non-metal pipes are extensively used (Gonthier 2012; Kalantar Mehrjerdi et al. 2020; Raymond et al. 2011). However, copper pipes are more widely used in sandbox tests because they have three benefits: (1) their low thermal resistance enables the thermocouples to monitor the real temperature of circulating fluid inside the pipes, (2) they allow for the choice of different diameters of pipes to make experimental, scaled-down U-shapes (Gu and O'Neal 1998), and (3) their coefficients of thermal expansion (about 16.8 × 10^−6^/°C) is similar to the cement-based grout (15–20 × 10^−6^/°C) (Meyers 1940). This is because if the difference in coefficient of thermal expansion between the grout and pipe is great, the repeated cycles of heating and cooling of BHE can lead to debonding and the formation of gaps between the grout and pipe, which hinder effective heat transfer within the borehole (Allan 2000). Such gaps lead to uneven heat transfer along the pipe and borehole walls (Philippacopoulos and Berndt 2001), complicating result interpretation and diminishing test accuracy.

### Circulating fluid and thermocouple

The circulating fluid, or heat carrier fluid, is the liquid medium that transfers heat within the heat exchanger pipe in the BHE system. During the test, the circulating water is heated to a specific temperature and injected into the heat exchanger pipe by the water pump from the water tank, and the velocity of the circulating fluid is regulated by the flow meter, as illustrated in Fig. 1. The circulating fluid used in field applications is generally a mixture of antifreeze and anti-corrosion agents, diluted with water to a concentration of 20%–25% (Bucci et al. 2018; Emmi et al. 2017). Similarly, Yu et al. (2004, 2008) employed a fluid mixture of 70% water and 30% ethylene glycol for cooling storage in the sandbox tests. However, in most reported sandbox tests, water is commonly used as the circulating fluid since the system usually does not operate below freezing point.

The thermocouple is utilized to monitor the temperatures of the circulating fluid, grouting material, and filling material (Reuss et al. 1997; Shirazi and Bernier 2014; Zhang et al. 2020). From the reported sandbox tests, Type-K (Ma et al. 2021), Type-T (Cimmino and Bernier 2015; Kramer et al. 2015; Li et al. 2020), and Type PT100 (Ma et al. 2023; Sezer et al. 2023) thermocouples with waterproof and anticorrosion features are widely employed. Protective shrink tubes are used to encase the thermocouples to protect them from damage during the installation (Kramer et al. 2015; Li et al. 2019). Thermocouples can be inserted into the inlet and outlet of the pipe to measure the temperature of the circulating fluid. When the grouting material is installed in the sandbox, thermocouples are embedded in the grouting material to measure its temperature variations. To monitor the temperatures of the filling material, thermocouples can be uniformly distributed in the filling material along the radial direction and the depth direction at specific intervals, as illustrated in Fig. 1.

### Groundwater flow

Typically, actual BHEs operate at depths less than 200 m, where groundwater is commonly found (Aresti et al. 2018; Fan et al. 2013; Florides and Kalogirou 2007; Haehnlein et al. 2010). While groundwater flow has been proven to significantly affect BHE performance, only a few studies have incorporated the groundwater flow into the sandbox test (Li et al. 2020; Ma et al. 2021; Wang et al. 2023). In these tests, groundwater flow was generally simulated in accordance withDarcy’s Law, provding a fundamental framework for describing fluid flow through porous media.

**Fig. 7:**
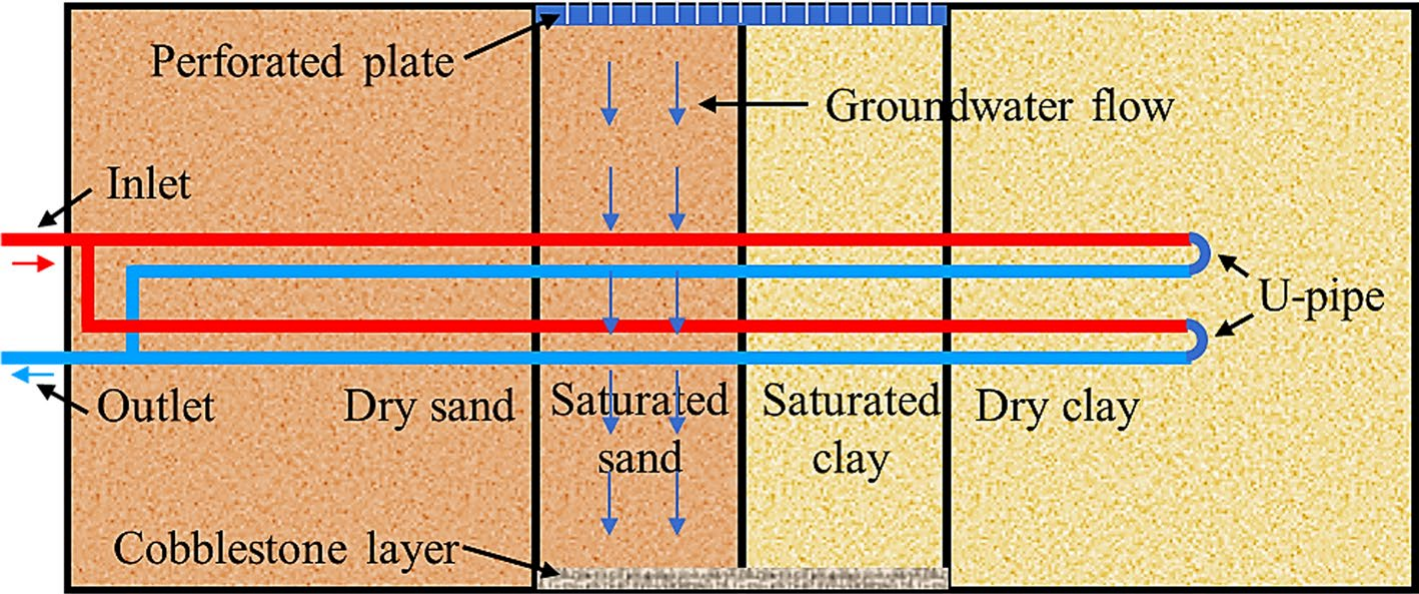
Schematic configuration of sandbox frame (modified from Li et al. (2020))

Among the reported studies, Li et al. (2020) developed a sandbox in which groundwater seepage was generated using a pluviation-based infiltration approach. Specifically, a perforated plate with small holes was located at the top center of the sandbox frame, and it worked as the pluviation system to “rain” water uniformly inside the sand and clay. At the bottom center of the sandbox frame, a cobblestone layer was used to preserve the sand and clay, as illustrated in Fig. 7. During the tests, an upper water tank flowed the water evenly inside the layered sand and clay through the small holes in the perforated plate. With gravity and water seepage, the water penetrated through the sand and reached the bottom cobblestone layer. Finally, the water returned to the lower water tank for the next circulating cycle. According to Darcy’s law, the water flow rate was adjusted by changing the heights of the upper and lower water tanks. Using this configuration, seepage velocities of 0.94 m/d and 1.11 m/d were implemented.

**Fig. 8:**
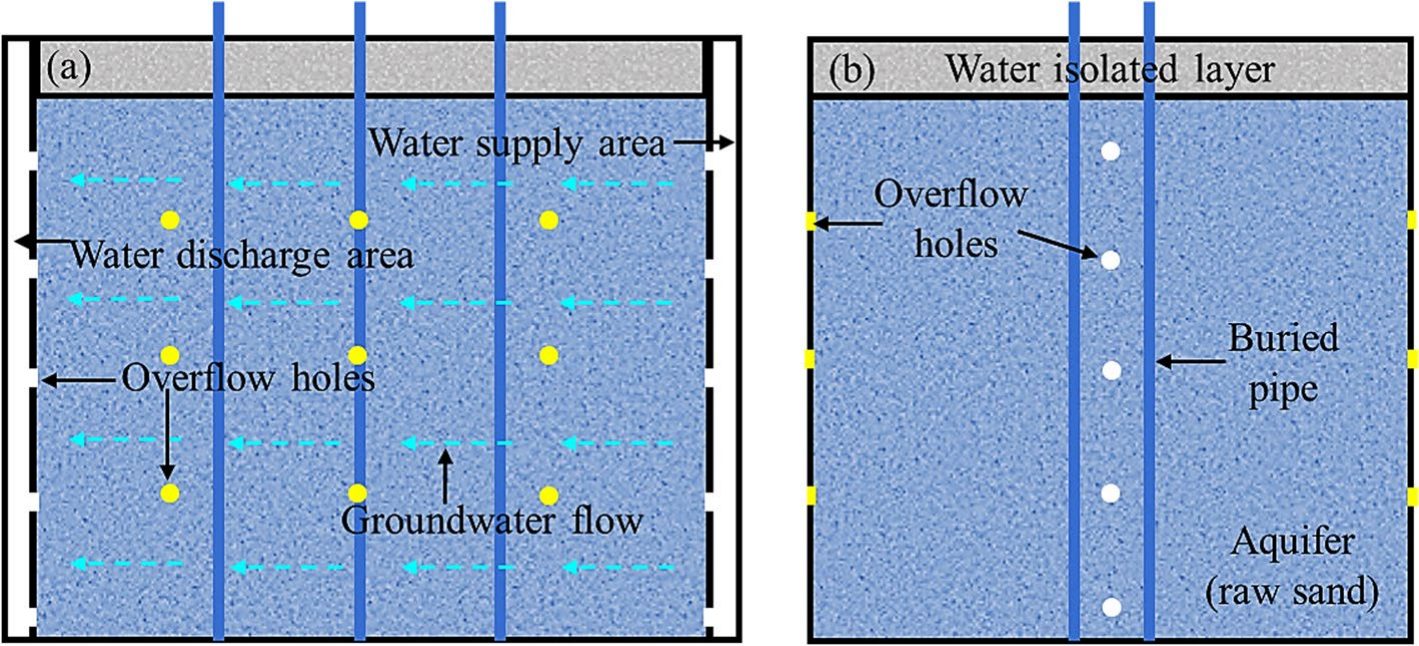
**a** front view and **b** side view of sandbox frame (modified from Ma et al. (2021))

Similar to the study by Li et al. (2020), a seepage area was created by using upper and lower water tanks in a sandbox developed by Wang et al.(2023). Five seepage velocities from 1 × 10^–7^ to 1 × 10^–5^ m/s were achieved, and the effects of seepage velocity on the heat transfer rate and pipe outlet temperature were investigated after a 30-day heat injection operation. Results indicated that as seepage velocity increased, the heat transfer rate significantly improved while the pipe outlet temperature decreased. This effect is attributed to the enhanced convection heat transfer capacity at higher seepage velocities, which effectively dissipates more heat from the soil, reducing heat accumulation around the borehole. Building on a similar Darcy's seepage concept but adopting a different control strategy, Ma et al. (2021) simulated the groundwater by injecting water from the top of the designated supply area and allowing it penetrated through the raw sand within the whole aquifer region to the water discharge area, as depicted in Fig. 8. During the test, the hydraulic difference between the water supply area and discharge area was adjusted by opening and closing the overflow holes (white dots and notches) at different heights. In addition, the water seepage velocity was regulated by opening and closing the overflow holes (yellow dots and notches) on the sidewalls. Using these water overflow holes, water supply, and discharge areas, a darcy velocity of seepage from 2.4–3.2 × 10^–7^ m/s to 2–3 × 10^–6^ m/s was developed. Temperature sensors were embedded at 0.5 m from the bottom of the sandbox in the middle of U-shape pipe to monitor the temperature of soil and seepage. By adjusting the seepage velocity, the researchers found that the range of thermal diffusion in the downstream changed. With the increase of the flow velocity, the migration speed of the temperature fronts accelerated, and the range of thermal diffusivity expanded. This can help reduce soil thermal accumulation and alleviate soil thermal interference effectively.

### Insulation layer 

The insulation layer can be applied at frame boundaries to minimize the influence of the surrounding environment on the temperature distribution within the sandbox. The thermal insulation is especially important when the sandbox is situated in an outdoor environment with large and uncontrollable temperature fluctuations (Li et al. 2018). In the reported sandbox tests, the most used materilas for insulation layers include styrofoam thermal panels, polystyrene insulation plates, thermal insulation mortars, and fiberglass insulation layers. For example, to create the fully thermally insulated testing conditions, Javadi et al. (2022) covered all the exterior sidewalls of the sandbox frame with styrofoam thermal panels and placed the polystyrene insulation plates for the four sections in the grouting material tests, as depicted in Fig. 4. Alternatively, thermal insulation mortars were attached to the inner sidewalls of a concrete sandbox frame developed by Li et al. (2017, 2019, 2018, 2020). In addition, to simulate the one-dimensional radial heat transfer mode alongside the heat exchanger pipes, insulation layers are often applied at the top and bottom of the sandbox frame to minimize the heat exchange between the two ends of the sandbox frame and the environment (Kramer et al. 2015; Shirazi 2012; Shirazi and Bernier 2014; Wang et al. 2013). For instance, Kramer et al. (2015) applied fiberglass insulation layers at the top and bottom of the test chamber for the sand and concrete thermal conductivity measurement to develop purely radial heat dissipation.

From a practical perspective, foam panels, polystyrene plates, and fiberglass layers are widely used as insulation layers on either the interior or exterior sidewalls of the sandbox frame. This is becausethey are more convenient to install and remove compared with insulation mortar. Furthoremore, to maintain a stable inlet temperature prior to heat exchange within the sandbox, the connecting hoses and pipes located outside the sandbox frame should be thermally insulated.

## Determination of sandbox size and experiment duration

As mentioned above, laboratory-scale sandbox tests were conducted to simulate the thermal behavior of the BHE system. By carefully controlling and isolating experimental parameters, these tests can evaluate the impact of specific factors on BHE performance. Such factors include, but are not limited to, the circulating fluid, grouting material, pipe configuration, borehole field layout, and groundwater seepage. These factors will induce discernible effects on BHE performance at diverse temporal and spatial scales. Therefore, a good understanding of the temporal and spatial scales relevant to the research purpose is essential prior to scaling down the BHE for sandbox tests.

From a temporal perspective, the thermal behavior of a BHE can be divided into two primary phases: short-term and long-term. The short-term involves the transient heat transfer within the borehole before it reaches a quasi-steady state. The duration typically extends to approximately 10 times *t*_b_ (where *t*_b_ = *r*_b_^2^/*α*_b_ is the characteristic time, *r*_b_ is the borehole radius, and *α*_b_ is the thermal diffusivity of the grouting material), where *t*_b_ is on the order of 1 h (Li et al. 2014a). During this phase, the borehole thermal resistance and heat capacity of the grouting material dominate the heat transfer process. The long-term involves the transients heat transfer between the borehole and the surrounding ground. The long-term usually extends to the lifetime of the BHE (up to 30 years), or even until the borehole wall temperature reaches a steady state. The characteristic time for the latter is 10 times *t*_*H*_ (where *t*_*H*_ = *H*^2^/*α*_g_ is the characteristic time, *H* is the borehole length, and *α*_g_ is the thermal diffusivity of the ground), which can be hundreds or even thousands of years (Li and Lai 2015). During this phase, while horizontal heat transfer between the borehole and the surrounding ground dominates, the influence of vertical heat transfer becomes increasingly significant over time. Additionally, factors such as multilayered geological conditions, groundwater flow, and surface boundary conditions exert a growing impact on the subsurface temperature distribution and the BHE performance. Furthermore, there is a critical time point within a borehole field, approximately *t*_*B*_ (where *t*_*B*_ = *B*^2^/(4*α*_g_) is the characteristic time, *B* is the borehole spacing, and *α*_g_ is the thermal diffusivity of the ground), which is typically on the order of a month. At this point, the thermal interaction between neighboring boreholes becomes significant (Li and Lai 2015). In summary, a clear understanding of these spatial scales is essential for designing tests of appropriate experimental duration.

When scaling sandbox setups, it is crucial to ensure that the sandbox accurately replicates the behavior of the actual BHE. To achieve this, two non-dimensional parameters—Reynolds number (*Re*) and Fourier number (*Fo*)—must be maintained equal between the sandbox test and the actual BHE. This ensures that the fluid flow and heat transfer characteristics in the sandbox closely match those in real-world conditions. The Reynolds number, defined as *Re* = *ρvL*/*μ* (where *ρ* is fluid density, *v* is flow velocity, *L* is the characteristic length, and *μ* is dynamic viscosity), helps determine the flow velocity within the pipe. By matching the *Re* between the sandbox and actual BHE, it guarantees that the model operates under the same flow regime as the real system. The Fourier number, defined as *Fo* = *αt/L*^*2*^, (where α is thermal diffusivity, *t* is time, and *L* is the characteristic length), is critical for scaling time and thermal diffusion effects appropriately between the sandbox and the actual BHE. Matching *Fo* ensures that thermal behavior, such as heat conduction, remains consistent regardless of size differences.

At this point, one can easily determine the experimental duration and dimensions of the BHE in the sandbox. Consider dimensional scaling factor (C_*l*_), defined as the ratio of a characteristic length of the actual BHE to the corresponding characteristic length of the BHE in the sandbox. By ensuring *Fo* remains consistent between the sandbox and the actual BHE, the time scaling factor (C_*t*_) can be calculated as the square of the dimensional scaling factor (i.e., C_*t*_ = C_*l*_^2^). Conversely, given a desired experimental duration, the necessary dimensional scaling factor can be determined. For example, if a 30-year BHE operation is to be mimicked within one month in the sandbox, a time scaling factor of 360 is needed. This requires the dimensions of the BHE to be scaled down by approximately 19 times in the sandbox. To maintain geometric similarity during scaling, it is suggested to proportionally reduce not only the borehole length but also the shank spacing and the diameters of the heat exchanger pipes. However, practically, it is extremely challenging to significantly reduce pipe diameters. Among sandbox tests, Cimmino and Bernier (2015) scaled down the pipe diameters to a minimum of 3.715 mm, while actual pipe diameter normally ranges from 20 to 40 mm (Gudni 2022; Li et al. 2014b; Rybach 2001, 2022; Zanchini and Jahanbin 2018). When further downsizing pipe diameter is impractical, alternative approaches, such as adjusting the aspect ratio, extending the experimental duration, or reducing the thermal conductivity of the filling material, can be considered to maintain a consistent *Fo* between the sandbox and the actual BHE.

Finally, when determining the sandbox size, it is essential to ensure that the sandbox is sufficiently large to prevent boundary effects from affecting the temperature distribution within the sandbox. This is aligned with real-world BHE applications, where the ground is often assumed to be semi-infinite without boundaries. A sandbox that is too small allows heat reaching the boundaries prematurely, causing reflections at boundaries and compromising the accuracy of the experimental results. As proposed by Claesson and Eskilson (1988), in the BHE operation, the temperature remains relatively constant beyond the far-field distance, *L* = (9*αt*)^1/2^ (where α is the ground thermal diffusivity and *t* is time). To minimize boundary effects, the sandbox dimensions are suggested to exceed this far-field distance. For instance, for a BHE operating in Ottawa sand (thermal diffusivity of 2 × 10^–7^ m^2^/s) (Cimmino and Bernier 2015) with adiabatic boundaries for one month, the suggested distance between the BHE and the sandbox boundary is more than 2.16 m. If the focus is solely on the temperature variations around the borehole wall, a distance of more than 1.08 m is recommended. This ensures that boundary effects are negligible, preserving the assumption of a semi-infinite medium for the ground during the sandbox test and enabling results that accurately replicate real-world heat transfer conditions.

## Existing problems and perspectives

The sandbox test utilizes a scaled-down testing rig that replicates the behavior of a full-size BHE, allowing researchers to simulate and analyze its performance in a cost-effective and time-efficient way. Previous sandbox tests have shown the following takeaways:

1. Multiple temperature sensors in sandbox setups can measure the temperature field during injection, providing high-resolution spatial and temporal data (Beier et al. 2011; Cimmino and Bernier 2015; Eslami Nejad and Bernier 2012; Zhang et al. 2020). For instance, Zhang et al. (2020) recorded the temperature profiles of the sandbox at different radius positions and depth during heat injection and extraction periods. The results show that during heat injection, higher heat source temperatures lead to greater soil temperature increases. Conversely, during heat extraction, higher heat sink temperatures result in smaller temperature reductions. During the heat injection or extraction process, temperature changes are more pronounced along the radial direction than the depth.
2. Sandbox tests can describe the seepage-temperature fields under controlled conditions, revealing the relationship between fluid flow and thermal dynamics. For example, Li et al. (2020) investigated the temperature distribution in the seepage area and found seepage affects the heat distribution of the pipe and the ground. The results show the seepage can enhance the heat transfer of borehole when the direction of seepage is vertical to the borehole by redistributing the heat within the ground to recovery the temperature.
3. Sandbox tests can be used to replicate subsurface conditions, enabling researchers to validate and verify numerical and analytical models for BHEs. The experimental data from sandbox tests can serve as a benchmark to compare and refine numerical and analytical models. For instance, the data derived from the sandbox test conducted by Beier et al. (2011) were widely used to validate numerical models by comparing numerical results with experimental findings, such as the ground temperature response (Guo et al. 2024a), the borehole wall temperature profiles (Guo et al. 2024a), and the inlet and outlet flow temperatures (Qian and Wang 2014). This dataset was also used to validate the software OpenGeoSys (Kolditz et al. 2016). Additionally, Cimmino and Bernier (2015) measured the borehole wall temperature and the net heat injection rate at a steady-state condition in a sandbox after a 168 h long test and calculated the experimental g-function. The results show the experimental g-function is highly consistent with the g-function obtained from the finite line source solution. These results affirm the effectiveness of sandbox experiments in optimizing BHEs by enabling precise measurements and aiding the development of predictive models.

Despite its advantages, the sandbox test encounters several limitations that impact its ability to accurately simulate BHE systems:

1. Limited dimensions and scaling challenges: Most dimensions of sandbox setups are insufficient to evaluate the long-term performance of BHEs. These tests are often restricted by space and time, which prevents them from replicating the full-scale dynamics of real systems. The dimensions of the sandbox components, such as the pipe diameter, shank spacing, and borehole dimension, do not always follow appropriate scaling rules. Consequently, the test results may not accurately reflect the three-dimensional heat distribution in real-world applications.
2. Temperature measurement limitations: The accuracy of temperature measurement in sandbox tests is a challenge. Traditional temperature sensors are typically placed at single points, providing limited thermal response data. This approach does not capture the spatial variation of temperatures along the borehole wall or the surrounding ground, and potentially misses critical insights into the heat distribution in BHEs.
3. Simplified ground conditions: Previous sandbox tests often fail to account for the complexity of real-world ground layers, which vary in porosity, moisture content, and permeability. These variations can have a significant impact on heat transfer and fluid movement, but they are not adequately reproduced in simplified sandbox environments.
4. Unidirectional seepage tests: In many existing sandbox tests, groundwater flow is modelled in a single direction, overlooking the more complex reality of multidirectional flow and variable hydraulic heads in natural aquifers. This can lead to an incomplete understanding of how groundwater seepage affects the BHE system’s thermal performance.
5. Absence of the geothermal gradient: One of the key characteristics of the subsurface environment is the geothermal gradient, which represents the increase in temperature with depth. Most sandbox tests do not incorporate this gradient, further reducing the realism of the tests.
6. Not applicable to deep geothermal energy applications: The deep borehole has considerably greater length-diameter ratios (e.g., 5714) (Morchio and Fossa 2019) than the shallow borehole (e.g. 778) (Guo et al. 2024b), making it hard to scale down the borehole into a sandbox by applying the scaling law. Additionally, deep geothermal systems involve high-temperature and high-pressure environments, which significantly affect hydraulic and heat transfer properties of geothermal fracture (Shu et al. 2020). However, the high-temperature and high-pressure environments are difficult to replicate in a sandbox under lab environments. As a result, while sandbox experiments excel at shallow-scale studies, their current design limits their use for deep geothermal applications.

To overcome these limitations and enhance the accuracy and reliability of sandbox tests, several improvements can be made:

1. Adhering to scaling rules: The dimensions of all components in the sandbox, such as pipe diameters, shank spacing, and borehole diameters, are suggested to follow scaling rules to ensure the tests more accurately reflect the behavior of full-scale BHE systems.
2. Prolonging test durations with low thermal conductivity filling materials: Using filling and grouting materials with low thermal conductivity can slow down the heat transfer process, extending test durations and enabling better assessment of long-term BHE performance.
3. Advanced temperature sensing techniques: Employing advanced temperature sensing technologies can significantly improve data accuracy. For example, distributed temperature sensing (DTS) using optical fibers can monitor temperature variations along the entire pipe wall, grouting materials, and backfilling materials (Joe et al. 2018; Ma et al. 2022; Mihailov 2012; Roriz et al. 2020). Wireless probes could be introduced to measure both the temperature and pressure inside the circulating fluids within the pipes, providing a more comprehensive understanding of system performance (Wilke et al. 2020).
4. 3D-printed ground models: To better replicate the variability of underground conditions, 3D printing technology can be employed to create ground models with different porosities and fractures (Kong et al. 2021). This method allows for a more realistic simulation of groundwater seepage under various conditions, improving the test’s accuracy.
5. Other accuracy improvement methods: During the tests, the external influences need to be minimized by preventing the sandbox from environmental distribution such as vibrations and temperature fluctuations. All sensors are required to be calibrated to minimize errors in data acquisition. Additionally, repetitive tests under identical conditions are necessary to confirm repeatability and ensure the reliability of sandbox tests. Furthermore, it is important to compare sandbox results with classical theoretical predictions (e.g., line source models), other validated experimental data, and results from established numerical models to ensure consistency of sandbox test results.

## Concluding remarks

The sandbox test utilizes a scaled-down testing rig that replicates the behavior of a full-size borehole heat exchanger (BHE), allowing researchers to simulate and analyze its performance in a cost-effective and time-efficient way for geothermal systems. The BHE is a complicated system, and the physical components of the sandbox test are similarly complex. This paper has reviewed the setups of the physical components of the sandbox test, including the sandbox frame, filling material, grouting material, heat exchanger pipe, circulating fluid, thermocouple, groundwater setup, and insulation layer. The determination of the sandbox dimensions and experiment duration were discussed. The main findings are listed as follows:

1. Most existing sandbox setups cannot assess the long-term performance of BHEs because of limited dimensions and non-compliance with scaling rules, which reduces testing accuracy. To address this limitation, we suggest extending the testing duration or using the filling material with low thermal conductivity to slow down the heat transfer process. Meanwhile, we recommend proportionally scaling down all components, including shank spacing, pipe diameter, and pipe length.

(2) The temperature measurement accuracy in sandbox tests remains limited. This is because conventional sensors typically capture thermal response at single points, which provides insufficient information on the overall thermal behavior of the borehole and surrounding ground and fails to reflect spatial temperature variations. We recommend adopting advanced temperature sensing techniques, such as optical fiber sensing to monitor temperature distributions alone the entire pipe and borehole walls, as well as wireless probes to track temperature and pressure variations in the circulating fluid.

(3) Existing sandbox tests overlooked heterogeneous ground layers with different varying porosities, moisture contents, and permeabilities. In addition, seepage tests were commonly limited to single-direction flow, ignoring multidirectional groundwater seepage and hydraulic head variations. We suggest using advanced fabrication techniques, such as 3D printing technique, to more realistically simulate layered ground conditions with diverse porosities and fracture networks, thereby enabling more accurate groundwater seepage modelling.
